# 

**Published:** 1889-09

**Authors:** 


					﻿OLD SERIES
Vok XXV
& 1855 o
NEW SERIES
Vol. VI.-No. 1.
® Journals.

W. F. WESTMORELAND, M.D.
H. V. M. MILLER, M.D., LL.D.
VIRGIL 0. HARDON, M.D.
F. W. McRAE, M.D., Man. & Pro.
Published ky james p.harrison&cq3
»__________ «Stlanta,ga. Affl
SUBSCRIPTION t 12.00 A YEAR
				

